# Imaging phenotyping using ^18^F-FDG PET/CT radiomics to predict micropapillary and solid pattern in lung adenocarcinoma

**DOI:** 10.1186/s13244-023-01573-9

**Published:** 2024-01-08

**Authors:** Linyi Zhou, Jinju Sun, He Long, Weicheng Zhou, Renxiang Xia, Yi Luo, Jingqin Fang, Yi Wang, Xiao Chen

**Affiliations:** 1 Department of Nuclear Medicine, Daping Hospital, Army Medical University, Chongqing, China; 2 Department of Ultrasound, Daping Hospital, Army Medical University, Chongqing, China; 3 Chongqing Clinical Research Center for Imaging and Nuclear Medicine, Chongqing, China

**Keywords:** Positron emission tomography, Computed tomography, Radiomics, Lung adenocarcinoma, Machine learning

## Abstract

**Objectives:**

To develop and validate a machine learning model using ^18^F-FDG PET/CT radiomics signature and clinical features to predict the presence of micropapillary and solid (MP/S) components in lung adenocarcinoma.

**Methods:**

Eight hundred and forty-six patients who underwent preoperative PET/CT with pathologically confirmed adenocarcinoma were enrolled. After segmentation, 1688 radiomics features were extracted from PET/CT and selected to construct predictive models. Then, we developed a nomogram based on PET/CT radiomics integrated with clinical features. Receiver operating curves, calibration curves, and decision curve analysis (DCA) were performed for diagnostics assessment and test of the developed models for distinguishing patients with MP/S components from the patients without.

**Results:**

PET/CT radiomics-clinical combined model could well distinguish patients with MP/S components from those without MP/S components (AUC = 0.87), which performed better than PET (AUC = 0.829, *p* < 0.05) or CT (AUC = 0.827, *p* < 0.05) radiomics models in the training cohort. In test cohorts, radiomics-clinical combined model outperformed the PET radiomics model in test cohort 1 (AUC = 0.859 vs 0.799, *p* < 0.05) and the CT radiomics model in test cohort 2 (AUC = 0.880 vs 0.829, *p* < 0.05). Calibration curve indicated good coherence between all model prediction and the actual observation in training and test cohorts. DCA revealed PET/CT radiomics-clinical model exerted the highest clinical benefit.

**Conclusion:**

^18^F-FDG PET/CT radiomics signatures could achieve promising prediction efficiency to identify the presence of MP/S components in adenocarcinoma patients to help the clinician decide on personalized treatment and surveillance strategies. The PET/CT radiomics-clinical combined model performed best.

**Critical relevance statement:**

^18^F-FDG PET/CT radiomics signatures could achieve promising prediction efficiency to identify the presence of micropapillary and solid components in adenocarcinoma patients to help the clinician decide on personalized treatment and surveillance strategies.

**Graphical Abstract:**

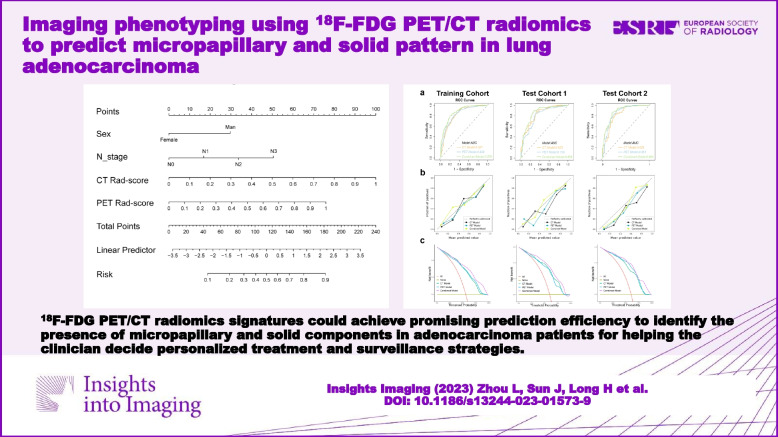

**Supplementary Information:**

The online version contains supplementary material available at 10.1186/s13244-023-01573-9.

## Key points

• ^18^F-FDG PET/CT radiomics signature is valuable to identify the presence of MP/S components in lung adenocarcinoma non-invasively.

• Gender and N stage are independent predictors of differentiation in patients with or without MP/S components.

• The nomogram integrating ^18^F-FDG PET/CT radiomics and clinical characteristics improves predictive performance.

**Supplementary Information:**

The online version contains supplementary material available at 10.1186/s13244-023-01573-9.

## Introduction

Lung cancer is the leading cause of death due to cancer globally, in which adenocarcinoma is the most common subtype [1]. Because of remarkable heterogeneity in adenocarcinoma, the International Association for the Study of Lung Cancer/American Thoracic Society/European Respiratory Society (IASLC/ATS/ERS) classified invasive lung adenocarcinoma into five subtypes, including lepidic, acinar, papillary, micropapillary, and solid [2]. Numerous studies reported that lung adenocarcinoma with micropapillary and solid (MP/S) predominant subtypes was related to poor prognosis [3, 4]. More importantly, patients with MP/S components accounting for 5% or greater would carry a high risk of early locoregional recurrence, who were performed with limited resection, but not lobectomy [5, 6]. Therefore, preoperative predictions of MP/S pattern in lung adenocarcinoma could help surgeons decide surgical strategies.

Biopsy is an optional method, but the sample obtained is only a portion of heterogeneous tumor, which cannot represent the whole tumor properties due to sampling error [7]. Non-invasive imaging can provide features of the whole tumors, containing structural and metabolic information spatiotemporally. ^18^F-fluorodeoxyglucose (FDG) positron emission tomography-computed tomography (PET/CT) has been widely applied to diagnose, stage, assess therapeutic efficacy, and predict the prognosis of lung cancer [8–11]. Emerging studies found that the tumor size, nodule type, ill-defined margin, and maximum standardized uptake value (SUVmax) derived from ^18^F-FDG PET/CT were associated with MP/S pattern in lung adenocarcinoma [3, 12–15]. However, these characteristics cannot reflect tumor heterogeneity. It is urgent to develop a more effective method to predict MP/S components in lung adenocarcinoma preoperatively.

Radiomics is the high-throughput extraction of quantitative medical image features, which provides detailed characteristics of tumor heterogeneity, offering a promising opportunity [16, 17]. Some studies found that quantitative CT features based on radiomics could distinguish MP/S components in lung adenocarcinoma. Nevertheless, the accuracy of the radiomics model was moderate [18, 19]. CT combining PET image information may increase the prediction accuracy of MP/S components in tumor. Recently, ^18^F-FDG PET radiomics has been reported to predict the metabolic status and heterogeneity in lung adenocarcinoma [20]. However, as far as we know, no predictive model has been reported to be developed based on PET/CT radiomics to predict MP/S components in lung adenocarcinoma.

In this study, we aimed to develop and validate a machine learning model by combining ^18^F-FDG PET/CT radiomics signature with clinical features to predict the presence of MP/S components in lung adenocarcinoma.

## Materials and methods

### Patients

**Fig. 1:**
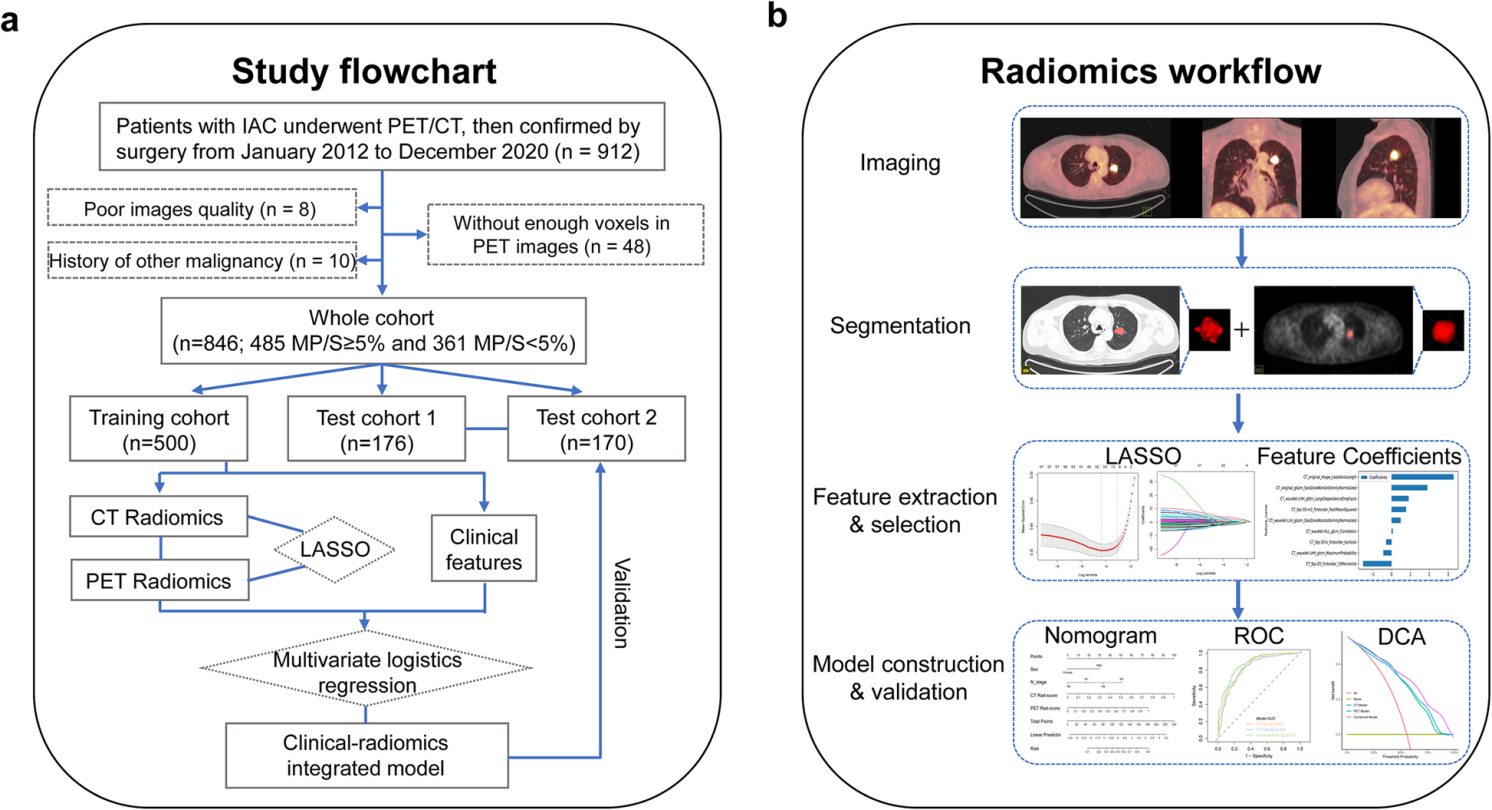
The workflow of this study. **a** Study flowchart. **b** Radiomics workflow

This retrospective study was approved by the Ethical Committee of Daping Hospital, Army Medical University (No.2022174), and the requirement for written informed consent was waived. Eight hundred and forty-six patients with pathologically confirmed invasive lung adenocarcinoma from January 2012 to December 2020 were enrolled. The classification of pathology is according to the 2011 IASLC/ATS/ERS classification of lung adenocarcinoma. Patients were classified into with MP/S group (MP/S component exceeded 5%) and without MP/S group (MP/S component less than 5%), based on the pathological analysis. The inclusion criteria were as follows: (1) pathologically confirmed invasive adenocarcinoma; (2) no radiotherapy, chemotherapy, or biopsy was received before ^18^F-FDG PET/CT scan; (3) within 2 weeks after PET/CT scan, the operation was done; and (4) patients were 18 years of age or older. The exclusion criteria were as follows: (1) poor image quality, (2) the lesion with 18F-FDG uptake values lower than or equal to the background, and (3) with history of other malignancy. Figure 1a shows the patient screening process.

### PET/CT imaging

Before scanning, all patients fasted > 6 h, with a blood glucose level < 10 mmol/L. Then, ^18^F-FDG was injected intravenously with the dose of 3.7 MBq/kg. Then, patients underwent PET/CT (Biograph 64 HD, Siemens Healthcare) scanning after 60 min from the vertex to the proximal legs. CT scan was firstly performed with the parameters of CT scan 120-kV voltage, 130-mA tube current, and 5-mm slice thickness. Whereafter, PET scan was conducted, scanning at 90 s/bed position and 4–6 bed positions. PET images were reconstructed by TrueD software on the Siemens workstation and fused with CT images.

### Image preprocessing, segmentation, and feature extraction

The volume of interest (VOI) was segmented by ITK-SNAP 3.8.0 software (www.itksnap.org). Two experienced nuclear medicine physicians (J.S. and L.Z.), without knowing the clinical and pathological results, semi-automatically delineated boundaries of the tumor to define the VOI (threshold = 40% SUVmax). Then, SUVmax and SUVavg of tumor VOIs were calculated in the PET images. For CT segmentation, VOI of the tumor was delineated on the lung window (window width = 1500 HU, window level =  − 700 HU). Then, the Pyradiomics 3.1.0 software was used to preprocess the images by resampling the isotropic voxel into 1 × 1 × 1 mm with linear interpolation. Finally, there are 1688 totally extracted radiomics features from each VOI including PET and CT images. To make features reproducible, an interclass correlation coefficient (ICC) greater than 0.75 indicates satisfactory agreement [21].

### Radiomics feature selection and model construction

The 846 patients were divided into training cohort, test cohort 1 and test cohort 2 at 6:2:2 randomly. Before selection, all features were normalized. A Mann–Whitney *U* validation was performed to remove the redundant features for initial feature selection. Then, regarding the dependence between features, a Pearson correlation analysis was done to exclude the features that the correlation coefficient was greater than 0.9. Subsequently, the least absolute shrinkage and selection operator (LASSO) algorithm was performed [22], and tenfold cross-validation was utilized to select the most distinguishable features. The rad-score was figured out by summing the selected features weighted by the corresponding coefficients. PET or CT rad-score means summing the selected PET or CT features weighted by the corresponding coefficients, respectively.

### Radiomics-clinical model construction

Gender, age, smoking, TNM stage, nodule type, air bronchogram, vacuole sign, pleural adhesion, shape (regular, irregular), maximum length of the tumor, interface (tumor lung interface: clear, unclear), SUVmax, SUVavg, and tumor metabolic volume (MTV) were collected. Nodule types were divided into three types, containing pure ground glass nodule (GGN), mixed GGN, and solid nodule based on thin-section unenhanced CT images. A pure GGN was defined as a nodule occupied by ground-glass opacity without solid regions. A mixed GGN was defined as a nodule that obscured underlying vascular signs and where < 50% of the nodule was observed at the mediastinal window. When more than 50% of a nodule was seen at the mediastinal window, a solid nodule was defined. The vacuole sign refers to a focal oval or round lucent area (typically < 5 mm). Two experienced nuclear medicine physicians (W.Z., R.X.) analyzed the PET/CT imaging features independently, without knowing the clinical data. These clinical features were compared to explore the distinguishing clinical features between two groups in training and test cohorts. Then, a multivariate logistic regression was used to develop a clinical-radiomics model to identify the presence of MP/S components in lung adenocarcinoma by combining rad-scores with selected clinical features. Receiver operating curves (ROCs) were adopted to assess the predictive performance of models to identify the presence of MP/S components in lung adenocarcinoma. Subsequently, based on the radiomics-clinical model, we established a nomogram. Hosmer–Lemeshow validation and calibration curves were performed to analyze the calibration of nomogram. Decision curve analysis (DCA) was adopted to evaluate the clinical practicability of these models.

### Statistics

Statistics were performed using the SPSS software (version 25.0, IBM Corp., Armonk, NY) and R 4.1.1 (http://www.R-project.org). The Kolmogorov–Smirnov and Levene tests were used to determine the normality and homogeneity of the variance, respectively. Independent *t*-tests or Mann–Whitney *U* tests were used to compare the continuous variables, and the chi-square tests or Fisher’s exact tests were used to compare the categorical variables. The Delong validation was used to compare the area under the curve (AUC) of the developed models. The level of significance for intergroup difference was set at *p* < 0.05.

## Results

### Patient characteristics

The baseline clinical characteristics of patients with or without MP/S components are shown in Table 1. Gender, smoking, T stage, N stage, nodule type, tumor size, SUVmax, SUVavg, and MTV exhibited significant difference between patients with or without MP/S in the training and test cohorts. In the training set, pleural adhesion (*p* = 0.014) and clear tumor–lung interface (*p* = 0.001) were more common in patients with MP/S components than those without MP/S, but not in the test cohorts (*p* > 0.05). There were no significant differences in age, air bronchogram, vacuole sign, and shape between patients with or without MP/S components (*p* > 0.05).

**Table 1:** Demographic and clinical characteristics of IAC patients with or without the presence of MP/S components

Clinical features	Training cohort (*n* = 500)			Test cohort 1 (*n* = 176)			Test cohort 2 (*n* = 170)		
	MP/S < 5%	MP/S ≥ 5%	*p* value	MP/S < 5%	MP/S ≥ 5%	*p* value	MP/S < 5%	MP/S ≥ 5%	*p* value
Gender			< 0.001			< 0.001			0.158
Male	76 (34.2%)	178 (64.0%)		24 (33.8%)	70 (66.7%)		31 (45.6%)	59 (57.8%)	
Female	146 (65.8%)	100 (36.0%)		47 (66.2%)	35 (33.3%)		37 (54.4%)	43 (42.2%)	
Age (years)	59.1 (8.70)	59.6 (9.50)	0.526	62.2 (9.08)	58.8 (9.64)	0.018	62.5 (9.52)	61.1 (9.33)	0.348
Smoking			< 0.001			< 0.001			0.034
Current or ever	67 (30.2%)	142 (51.1%)		18 (25.4%)	56 (53.3%)		22 (32.4%)	51 (50.0%)	
Never	155 (69.8%)	136 (48.9%)		53 (74.6%)	49 (46.7%)		46 (67.6%)	51 (50.0%)	
T stage			< 0.001			< 0.001			< 0.001
T1	163 (73.4%)	121 (43.5%)		52 (73.2%)	39 (37.1%)		49 (72.1%)	32 (31.4%)	
T2	53 (23.9%)	115 (41.4%)		17 (23.9%)	53 (50.5%)		15 (22.1%)	47 (46.1%)	
T3	4 (1.80%)	24 (8.63%)		1 (1.41%)	10 (9.52%)		4 (5.88%)	15 (14.7%)	
T4	2 (0.90%)	18 (6.47%)		1 (1.41%)	3 (2.86%)		0 (0.00%)	8 (7.84%)	
N stage			< 0.001			< 0.001			< 0.001
N0	200 (90.1%)	154 (55.4%)		65 (91.5%)	63 (60.0%)		60 (88.2%)	56 (54.9%)	
N1	10 (4.50%)	47 (16.9%)		3 (4.23%)	13 (12.4%)		4 (5.88%)	14 (13.7%)	
N2	10 (4.50%)	72 (25.9%)		3 (4.23%)	27 (25.7%)		3 (4.41%)	31 (30.4%)	
N3	2 (0.90%)	5 (1.80%)		0 (0.00%)	2 (1.90%)		1 (1.47%)	1 (0.98%)	
Nodule type			< 0.001			< 0.001			< 0.001
pGGN	20 (9.01%)	1 (0.36%)		9 (12.7%)	0 (0.00%)		7 (10.3%)	1 (0.98%)	
mGGN	78 (35.1%)	13 (4.68%)		21 (29.6%)	2 (1.90%)		22 (32.4%)	4 (3.92%)	
Solid	124 (55.9%)	264 (95.0%)		41 (57.7%)	103 (98.1%)		39 (57.4%)	97 (95.1%)	
Air bronchogram			0.587			0.322			0.421
( +)	55 (24.8%)	62 (22.3%)		24 (33.8%)	27 (25.7%)		17 (25.0%)	19 (18.6%)	
( −)	167 (75.2%)	216 (77.7%)		47 (66.2%)	78 (74.3%)		51 (75.0%)	83 (81.4%)	
Vacuole sign			0.707			0.806			0.325
( +)	30 (13.5%)	42 (15.1%)		11 (15.5%)	19 (18.1%)		8 (11.8%)	19 (18.6%)	
( −)	192 (86.5%)	236 (84.9%)		60 (84.5%)	86 (81.9%)		60 (88.2%)	83 (81.4%)	
Pleural adhesion			0.014			0.423			0.276
( +)	129 (58.1%)	192 (69.1%)		43 (60.6%)	71 (67.6%)		41 (60.3%)	71 (69.6%)	
( −)	93 (41.9%)	86 (30.9%)		28 (39.4%)	34 (32.4%)		27 (39.7%)	31 (30.4%)	
Shape			0.079			0.027			1
Regular	47 (21.2%)	41 (14.7%)		18 (25.4%)	12 (11.4%)		19 (27.9%)	28 (27.5%)	
Irregular	175 (78.8%)	237 (85.3%)		53 (74.6%)	93 (88.6%)		49 (72.1%)	74 (72.5%)	
Maximum length	2.00 [1.50; 2.60]	2.80 [2.10; 3.80]	< 0.001	2.00 [1.60; 2.55]	2.80 [2.20; 3.60]	< 0.001	2.05 [1.60; 2.70]	3.00 [2.30; 4.10]	< 0.001
Interface			0.001			0.327			0.096
Clear	75 (33.8%)	136 (48.9%)		27 (38.0%)	49 (46.7%)		25 (36.8%)	52 (51.0%)	
Unclear	147 (66.2%)	142 (51.1%)		44 (62.0%)	56 (53.3%)		43 (63.2%)	50 (49.0%)	
SUVmax	2.56 [1.23; 5.69]	9.39 [5.85; 14.3]	< 0.001	3.35 [1.65; 6.73]	9.30 [5.45; 15.3]	< 0.001	3.06 [1.30; 5.36]	10.2 [5.94; 14.0]	< 0.001
SUVavg	1.48 [0.76; 3.30]	5.48 [3.42; 8.55]	< 0.001	2.00 [0.98; 4.08]	5.55 [3.24; 8.39]	< 0.001	1.81 [0.87; 3.31]	6.20 [3.68; 8.38]	< 0.001
MTV	1.67 [1.07; 3.48]	4.61 [2.12; 10.8]	< 0.001	1.82 [1.17; 3.22]	4.33 [2.05; 8.85]	< 0.001	2.09 [0.89; 3.57]	4.63 [2.42; 14.4]	< 0.001

Medians (interquartile range) on behalf of maximum length, SUVmax, SUVavg, and MTV, because they did not comply with normal distribution

*p* < 0.05 showed significant difference

### Radiomics feature selection and signature construction

**Fig. 2:**
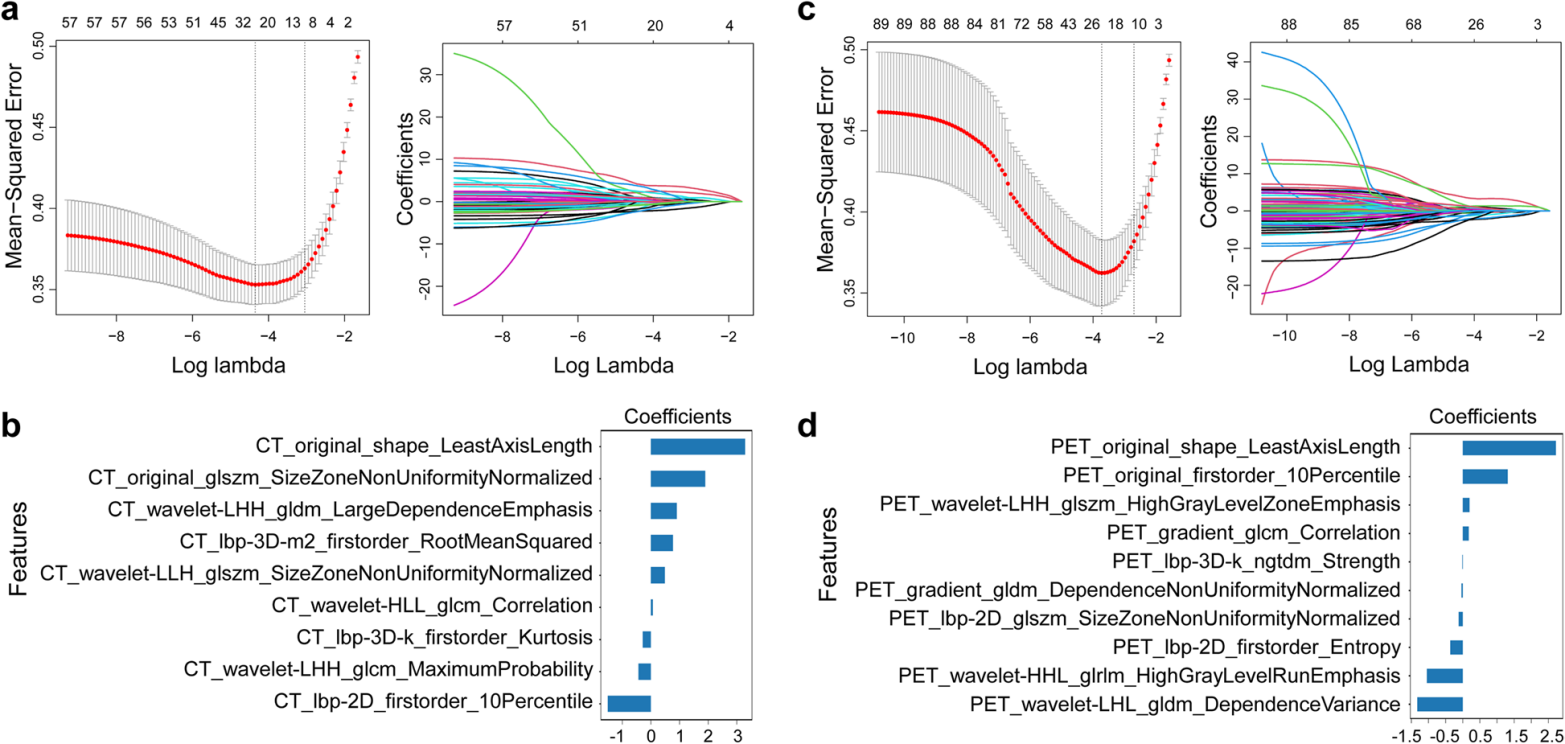
Radiomics feature selection. CT (**a**) and PET (**c**) radiomics features were selected by the LASSO model. The retained features after selection and their corresponding coefficients for CT (**b**) and PET (**d**) radiomics

**Fig. 3:**
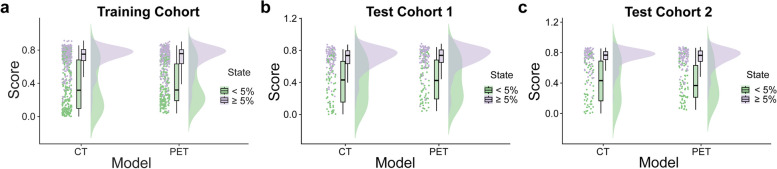
Raincloud plot visualizes the prediction probability of CT and PET radiomics signature. It shows the rad-score distribution of patients in training cohort (**a**) and test cohorts (**b**,** c**)

We totally extracted 1688 features from PET/CT images. The mean ICC value was 0.909, suggesting excellent inter-observer reproducibility and consistency of VOI drawing and feature extraction. *U*-test and Pearson correlation coefficient analysis were adopted to dimensionality reduction, resulting in 58 CT radiomics features and 89 PET radiomics features left. Subsequently, LASSO was performed to select the remaining features deeply. Ten radiomics features were retained for the PET model, while nine radiomics features were selected for the CT model (Fig. 2, Table S1, S2). Then, we constructed PET and CT predictive signatures by nonlinear SVM method, respectively. Figure 5a and Table 2 exhibit the performance of CT radiomics model and PET radiomics model. No significant differences between the CT model and PET model in training and test cohorts were found for distinguishing patients with or without MP/S components by Delong test analysis (*p* = 0.8783 in training cohort, *p* = 0.1892 in test cohort 1, *p* = 0.4189 in test cohort 2). The radiomics feature score obtained in SVM model of each patient was seen as rad-score. Raincloud plot showed the rad-score distributions of the patients in training and test cohorts, suggesting that patients with MP/S components had higher rad-score than those without MP/S components, with a discriminant ability (Fig. 3).

**Table 2:** The performance of three models in training and test cohorts

	AUC (95%CI)	SEN (%)	SPE (%)	ACC (%)	PPV (%)	NPV (%)
Training cohort						
PET model	0.829 (0.794–0.866)	81.65	71.17	77.00	78.01	75.60
CT model	0.827 (0.791–0.865)	58.56	94.24	78.40	74.01	89.04
Combined model	0.870 (0.840–0.902)	79.86	80.18	80.00	83.46	76.07
Test cohort 1						
PET model	0.799 (0.732–0.868)	84.76	69.01	78.41	80.18	75.38
CT model	0.833 (0.772–0.893)	87.62	67.60	79.55	78.69	80.00
Combined model	0.859 (0.800–0.918)	89.52	73.24	82.95	83.19	82.54
Test cohort 2						
PET model	0.854 (0.791–0.917)	90.20	70.59	82.35	82.14	82.76
CT model	0.829 (0.763–0.895)	83.33	72.06	78.82	81.73	74.24
Combined model	0.880 (0.826–0.934)	86.27	79.41	83.53	86.27	79.41

*AUC* area under the curve, *95% CI* 95% confidence interval, *SEN* sensitivity, *SPE* specificity, *ACC* accuracy, *PPV* positive predictive value, *NPV* negative predictive value

### Construction and validation of the nomogram

**Fig. 4:**
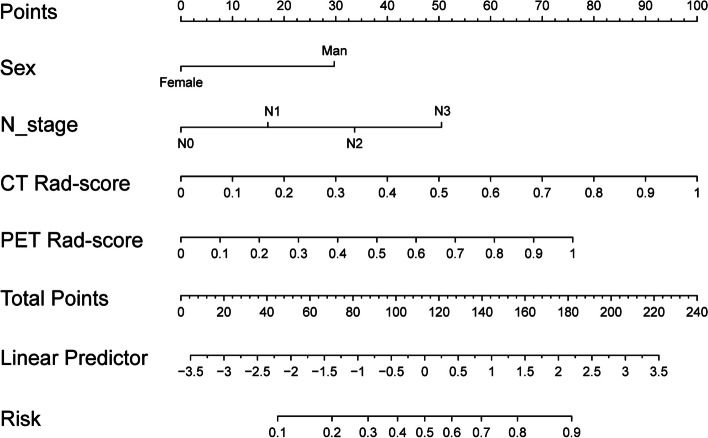
Nomogram based on PET/CT rad-score and clinical features

**Fig. 5:**
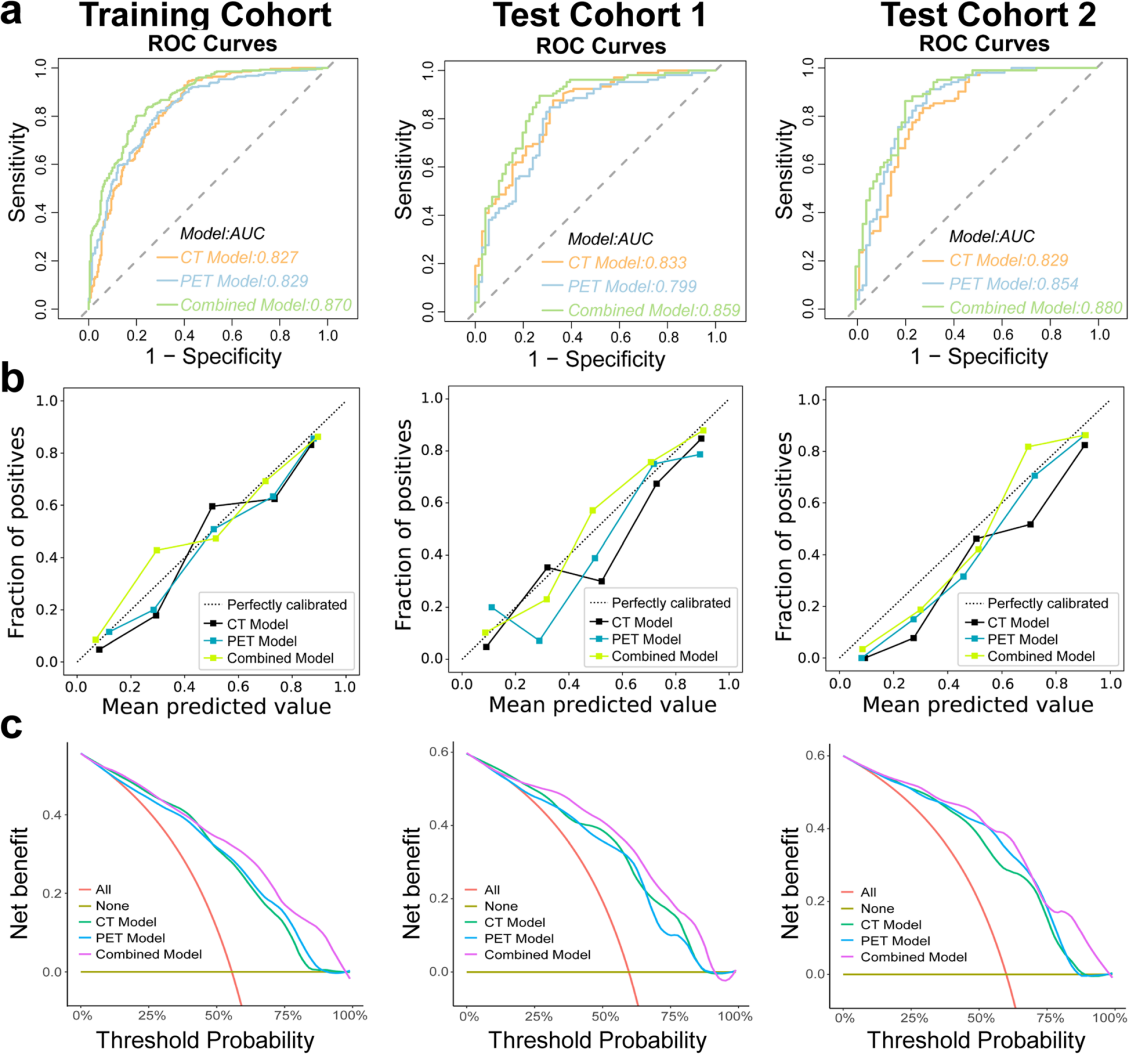
Diagnostic assessment and test of three models for distinguishing the patients with MP/S components from the patients without. ROC analysis (**a**), calibration curves (**b**), and decision curve analysis (**c**) of three models for identifying the presence of MP/S components in training and test cohorts

In combination with clinical variables, we further developed a combined model by logistic regression. The gender and N stage were independent predictors of differentiation in patients with or without MP/S components in the clinical-radiomics combined model by multivariate logistic regression analysis (Table S3). Figure 4 shows the nomogram based on rad-score and clinical features. The results revealed that radiomics-clinical combined model could well distinguish patients with MP/S components from those without MP/S components (AUC = 0.87), which performed better than PET radiomics model (AUC = 0.829, *p* < 0.05) or CT radiomics model (AUC = 0.827, *p* < 0.05) in training cohort. In test cohorts, the radiomics-clinical combined model performed better than the PET radiomics model in test cohort 1 (AUC = 0.859 vs 0.799, *p* < 0.05) and CT radiomics model in test cohort 2 (AUC = 0.880 vs 0.829, *p* < 0.05) (Fig. 5a, Table S4). The calibration curve indicated good coherence between all model prediction and the actual observation in training and test cohorts (Fig. 5b, *p* > 0.05). Subsequently, DCA was adopted to evaluate the clinical application of the three developed models. The results of DCA revealed PET/CT radiomics-clinical model exerted the highest clinical benefit to distinguish patients with MP/S from those without MP/S components (Fig. 5c).

## Discussion

Recently, lung adenocarcinoma with or without MP/S components has been drawing attention, which is associated with poor prognosis, and can influence surgical strategies [3, 23–26]. Therefore, it is important to predict the presence of MP/S components in lung adenocarcinoma preoperatively for optimal surgical strategies and whether to receive aggressive postoperative adjuvant therapy. Herein, we successfully developed a PET/CT radiomics signature and constructed a model by combining radiomics with clinical features to distinguish patients with MP/S components from those without MP/S components, which exhibited good performance. These findings suggest that the method to identify the presence of MP/S components by integrating PET/CT radiomics and clinical features could be potentially feasible in clinics.

Accumulating studies found that MP/S subtypes manifested as predictors for higher aggressive and worse prognosis [3, 24, 25]. Our results showed that the presence of MP/S components in lung adenocarcinoma was significantly associated with pleural invasion (*p* = 0.014) and metastasis of lymph node (*p* < 0.001), which were consistent with the previous studies [27–29]. Furthermore, with the increase of the use of sublobar resection, the optimal strategy for early-stage NSCLC patients is essential. Based on the results of a prospective multi-institutional study on the relationship between radiologic and pathologic findings in peripheral lung cancer, the general indication for sublobar resection in cases with radiological invasive lesions is a lesion size ≤ 2 cm. But some reports also demonstrated small (≤ 2 cm) early-stage lung adenocarcinoma patients with MP/S components accounting for 5% or greater would carry a high risk of early locoregional recurrence, who were performed with limited resection, but not lobectomy [5, 6]. In addition, in inoperable lung adenocarcinoma, determining the presence of MP/S is also important because biopsy may not reflect all features of the tumor due to tumor heterogeneity [18]. Therefore, preoperative predictions of MP/S components in lung adenocarcinoma could help surgeons decide on personalized treatment and surveillance strategies.

So far, few researches have studied the imaging-based prediction of MP/S components, which were all subjective studies based on qualitative CT and PET/CT variables. The results showed radiologic characteristics like tumor size > 2.5 cm, a solid nodule, and high ^18^F-FDG uptake were useful to detect the presence of MP/S components [3, 12, 30, 31]. In our study, patients with MP/S components showed higher ^18^F-FDG uptake, solid-predominant tumor, and larger tumor size than those without, which was consistent with the previous studies. Moreover, due to the aggressive behavior of MP/S patterns, tumors with the presence of MP/S components were related to lymphatic invasion. Therefore, lymph node involvement was more common in tumors with MP/S components than those without MP/S components [32, 33]. Our results showed the MP/S < 5% with a tendency to have a lower TNM stage, whereas the MP/S > 5% with more frequent lymph node metastasis. As for demographics, patients with MP/S patterns were found to be associated with males [3, 27], which was confirmed by our results.

Allowing for intratumor heterogeneity, radiomics containing abundant quantitative medical imaging features can provide a more detailed characterization of tumor heterogeneity, which reflects comprehensive quantification of disease phenotypes [17, 34]. Emerging studies found that quantitative CT features based on radiomics could distinguish MP/S components in lung adenocarcinoma. Nevertheless, the accuracy of the CT radiomics model was moderate [18, 19]. Adding metabolic information to CT images may increase the prediction accuracy of MP/S components in lung adenocarcinoma [35]. In our study, the performance of CT and PET radiomics models was good. Further, we established PET/CT radiomics-clinical combined model, including gender, N stage, CT rad-score, and PET rad-score. The combined model performed better than CT or PET radiomics models to identify the presence of MP/S components. Then, we adopt two test cohorts to validate the performance of the developed models. The performance of developed models in the two test cohorts remained stable, which indicated good generalizability of these models. Taken together, ^18^F-FDG PET/CT radiomics-clinical model could identify the presence of MP/S components in lung adenocarcinoma with high performance.

This study had some limitations. First, this was a retrospective, single-center study, which was limited by biases like incomplete data acquisition and patient selection. Prospective, multi-center study is necessary in the future. Second, external validation was not performed. However, we conducted internal validation using two test cohorts. The performance of developed models in the two test cohorts remained stable, which indicated good generalizability of these models. Third, the indication for sublobar resection in cases with a tumor size is less than 2 cm in general. But in this study, part of the patients with MP/S components enrolled in our study exceeded 2 cm, which may influence the results of the radiomics analysis. Study enrolling patients with tumor size eligible for sublobar resection is further needed.

In conclusion, we developed an optimal PET/CT radiomics signature and constructed a model to identify the presence of MP/S components in lung adenocarcinoma by combining radiomics with clinical features, with a good performance. This study suggests ^18^F-FDG PET/CT radiomics signatures could achieve promising prediction efficiency to identify the presence of MP/S components in lung adenocarcinoma patients to help the clinician decide on personalized treatment and surveillance strategies.

### Supplementary Information

**Additional file 1: Table S1.** The selected most predictive subset of feature and the corresponding coefficients in CT model. **Table S2.** The selected most predictive subset of feature and the corresponding coefficients in PET model. **Table S3.** Multivariate logistic analysis of the extracted clinical features for the presence of MP/S components. **Table S4.** Delong test for comparation of AUCs of the developed models.

## Data Availability

The datasets analyzed during the current study are available from the corresponding author upon reasonable request.

## Abbreviations

AUC: Area under the curve
DCA: Decision curve analysis
FDG: ^18^F-fluorodeoxyglucose
ICC: Interclass correlation coefficient
LASSO: Least absolute shrinkage and selection operator
MP/S: Micropapillary and solid
MTV: Metabolic tumor volume
PET/CT: Positron emission tomography-computed tomography
ROC: Receiver operating curve
SUVmax: Maximum standardized uptake value
VOI: Volume of interest
