# Supplementary material for: Imaging phenotyping using 18F-FDG PET/CT radiomics to predict micropapillary and solid pattern in lung adenocarcinoma
Source: Insights Imaging. 2024 Jan 8;15:5. doi: 10.1186/s13244-023-01573-9 (PMC10772036; doi:10.1186/s13244-023-01573-9)
Supplement: Supplementary file 1 — Additional file 1: Table S1. The selected most predictive subset of feature and the corresponding coefficients in CT model. Table S2. The selected most predictive subset of feature and the corresponding coefficients in PET model. Table S3. Multivariate logistic analysis of the extracted clinical features for the presence of MP/S components. Table S4. Delong test for comparation of AUCs of the developed models. [file 13244_2023_1573_MOESM1_ESM.docx]

**Imaging phenotyping using ^18^F-FDG PET/CT radiomics to predict micropapillary and solid pattern in lung adenocarcinoma**

**ELECTRONIC SUPPLEMENTARY MATERIAL**

**Table S1.** The selected most predictive subset of feature and the corresponding coefficients in CT model.

| Features | Coefficient |
| --- | --- |
| CT_lbp-2D_firstorder_10Percentile | -1.50777 |
| CT_wavelet-LHH_glcm_MaximumProbability | -0.43337 |
| CT_lbp-3D-k_firstorder_Kurtosis | -0.28491 |
| CT_wavelet-HLL_glcm_Correlation | 0.067824 |
| CT_wavelet-LLH_glszm_SizeZoneNonUniformityNormalized | 0.488254 |
| CT_lbp-3D-m2_firstorder_RootMeanSquared | 0.771335 |
| CT_wavelet-LHH_gldm_LargeDependenceEmphasis | 0.904726 |
| CT_original_glszm_SizeZoneNonUniformityNormalized | 1.89792 |
| CT_original_shape_LeastAxisLength | 3.289613 |

**Table S2.** The selected most predictive subset of feature and the corresponding coefficients in PET model.

| Features | Coefficient |
| --- | --- |
| PET_wavelet-LHL_gldm_DependenceVariance | -1.32777 |
| PET_wavelet-HHL_glrlm_HighGrayLevelRunEmphasis | -1.04585 |
| PET_lbp-2D_firstorder_Entropy | -0.36404 |
| PET_lbp-2D_glszm_SizeZoneNonUniformityNormalized | -0.11967 |
| PET_gradient_gldm_DependenceNonUniformityNormalized | -0.03855 |
| PET_lbp-3D-k_ngtdm_Strength | -0.01444 |
| PET_gradient_glcm_Correlation | 0.172793 |
| PET_wavelet-LHH_glszm_HighGrayLevelZoneEmphasis | 0.195616 |
| PET_original_firstorder_10Percentile | 1.313173 |
| PET_original_shape_LeastAxisLength | 2.718833 |

**Table S3.** Multivariate logistic analysis of the extracted clinical features for the presence of MP/S components.

| Variable | OR (95% CI) | P-value |
| --- | --- | --- |
| Gender | 0.343 (0.153, 0.733) | 0.007 |
| Smoking | 1.275 (0.586, 2.888) | 0.548 |
| T stage | 1.280 (0.856, 1.955) | 0.240 |
| N stage | 1.590 (1.141, 2.272) | 0.008 |
| Nodule type | 1.855 (0.906, 3.974) | 0.100 |
| Pleural adhesion | 1.425(0.813, 2.539) | 0.221 |
| Maximum length | 1.056 (0.751, 1.491) | 0.755 |
| Interface | 0.655 (0.383, 1.115) | 0.120 |
| SUVmax | 1.010 (0.805, 1.296) | 0.936 |
| SUVavg | 1.004 (0.672, 1.449) | 0.984 |
| MTV | 0.993 (0.972, 1.020) | 0.529 |

95% CI, 95% confidence interval

**Table S4.** Delong test for comparation of AUCs of the developed models.

| Model | Z | P |
| --- | --- | --- |
| Training Cohort |  |  |
| CT model vs PET model | -0.1531 | 0.8783 |
| CT model vs Combined model | -3.8700 | 0.0001 |
| PET model vs Combined model | -3.6806 | 0.0002 |
| Test Cohort 1 |  |  |
| CT model vs PET model | 1.3131 | 0.1892 |
| CT model vs Combined model | -1.3595 | 0.1740 |
| PET model vs Combined model | -2.8908 | 0.0038 |
| Test Cohort 2 |  |  |
| CT model vs PET model | -0.8083 | 0.4189 |
| CT model vs Combined model | -2.5481 | 0.0108 |
| PET model vs Combined model | -1.0936 | 0.2741 |
